# Controls on bacterial and archaeal community structure and greenhouse gas production in natural, mined, and restored Canadian peatlands

**DOI:** 10.3389/fmicb.2013.00215

**Published:** 2013-07-31

**Authors:** Nathan Basiliko, Kevin Henry, Varun Gupta, Tim R. Moore, Brian T. Driscoll, Peter F. Dunfield

**Affiliations:** ^1^Department of Geography, University of Toronto MississaugaMississauga, ON, Canada; ^2^Max-Planck-Institute for Terrestrial MicrobiologyMarburg, Germany; ^3^Department of Geography, University of UtahSalt Lake City, UT, USA; ^4^Department of Geography, McGill UniversityMontreal, QC, Canada; ^5^Department of Natural Resource Sciences, McGill UniversitySte. Anne de Bellevue, QC, Canada; ^6^Department of Biological Sciences, University of CalgaryCalgary, AB, Canada

**Keywords:** archaea, bacteria, carbon dioxide, decomposition, methane, methanogen

## Abstract

Northern peatlands are important global C reservoirs, largely because of their slow rates of microbial C mineralization. Particularly in sites that are heavily influenced by anthropogenic disturbances, there is scant information about microbial ecology and whether or not microbial community structure influences greenhouse gas production. This work characterized communities of bacteria and archaea using terminal restriction fragment length polymorphism (T-RFLP) and sequence analysis of 16S rRNA and functional genes across eight natural, mined, or restored peatlands in two locations in eastern Canada. Correlations were explored among chemical properties of peat, bacterial and archaeal community structure, and carbon dioxide (CO_2_) and methane (CH_4_) production rates under oxic and anoxic conditions. Bacteria and archaea similar to those found in other peat soil environments were detected. In contrast to other reports, methanogen diversity was low in our study, with only 2 groups of known or suspected methanogens. Although mining and restoration affected substrate availability and microbial activity, these land-uses did not consistently affect bacterial or archaeal community composition. In fact, larger differences were observed between the two locations and between oxic and anoxic peat samples than between natural, mined, and restored sites, with anoxic samples characterized by less detectable bacterial diversity and stronger dominance by members of the phylum *Acidobacteria*. There were also no apparent strong linkages between prokaryote community structure and CH_4_ or CO_2_ production, suggesting that different organisms exhibit functional redundancy and/or that the same taxa function at very different rates when exposed to different peat substrates. In contrast to other earlier work focusing on fungal communities across similar mined and restored peatlands, bacterial and archaeal communities appeared to be more resistant or resilient to peat substrate changes brought about by these land uses.

## Introduction

Northern peatlands are important long-term sinks of atmospheric carbon dioxide (CO_2_) due to net imbalances between primary production and heterotrophic mineralization of soil organic matter (peat) and plant litters (Roulet et al., 2007). They are also persistent sources of methane (CH_4_) due to waterlogging of soil profiles that helps sustain methanogenesis. Soil microorganisms, including bacteria and archaea, are largely responsible for the production of both of these greenhouse gases through decomposition processes. However, we do not fully understand the factors that control microbial community structure at these sites, nor can we yet make meaningful linkages between diversity and activities that ultimately result in greenhouse gas emissions (Andersen et al., 2013).

Microbial community controls may be particularly important to greenhouse gas flux dynamics in sites that are commercially mined (or “cutover”) for horticultural substrates and soil amendments (Andersen et al., 2013). In these sites the microbial environment is dramatically altered through removal of newly-formed peat and exposure of biorecalcitrant peat that is thousands of years old and the resulting hydrological and plant community alterations (Andersen et al., 2006; Basiliko et al., 2007). Depending on the method of peat extraction, natural restoration of mined peatlands can lead to the relatively rapid formation of new peat (Robert et al., 1999). However, active restoration as a tool to return peatlands mined using contemporary methods to sinks for CO_2_ and small sources of CH_4_ has produced variable results (Tuittila et al., 1999; McNeil and Waddington, 2003; Marinier et al., 2004; Waddington et al., 2010). Restoration efforts likely can set peatlands on a trajectory toward renewed carbon sequestration, however because this is a process that would subsequently takes thousands of years, there is a clear trade-off between loss of contemporary carbon stocks and provision of peat as a natural resource. Croft et al. (2001) first demonstrated that numbers of cultivatable bacteria were reduced after mining and Glatzel et al. (2004) later suggested that understanding the microbial role in both the impacts of mining and the effectiveness of restoration is essential.

In restored or revegetated mined peatlands, functional microbial fingerprints using physiological profiling techniques have linked substrate utilization abilities to the newly established vegetation on peatland surfaces (Artz et al., 2008; Yan et al., 2008). Previous work in mined and/or restored peatlands has also explored the role of decomposer community structure. Litter chemistry and plant communities have been linked to changes in fungal communities and carbon loss in mined peat surfaces recently colonized by different plant functional groups (Artz et al., 2007; Trinder et al., 2008, 2009). However, recent work has suggested that bacterial activities and numbers predominate over those of fungi across natural North American peatlands, including non-saturated surface peat of acidic bogs and poor fens (Winsborough and Basiliko, 2010; Lin et al., 2012; Myers et al., 2012), although mining and restoration impacts on bacterial communities have not been extensively studied. Across other non-impacted peat environments it has been shown that bacterial and archaeal decomposer communities can be quite similar despite different vegetation and litter chemistry and even when exhibiting different rates of activity (Kim et al., 2008; Preston et al., 2012). Given the importance of wetlands in global CH_4_ emissions, methanogenic archaea in peatlands have been the focus of study for some time (e.g., Williams and Crawford, 1984; Hales et al., 1996; Basiliko et al., 2003; Bräuer et al., 2006), including sites that have been drained for forestry and reflooded (Galand et al., 2005b; Juottonen et al., 2012). Restoration of peatlands used for forestry in Finland re-established methanogen communities similar to those in undisturbed sites, though methanogen abundance and CH_4_ emissions rates remained lower, despite generally similar water table positions (Juottonen et al., 2012). However, the impacts of horticultural mining and subsequent restoration on methanogens remain unknown.

The objectives of this work were to characterize communities of bacteria and archaea across eight natural, mined, or restored peatlands in two eastern Canada locations using terminal restriction fragment length polymorphism (T-RFLP) fingerprinting combined with sequence analyses of small sub-unit ribosomal RNA and methyl-coenzyme M reductase (*mcrA*) gene fragments. Correlations were explored among chemical properties of peat that are altered through land-use changes, bacterial and archaeal community structure, and CO_2_ and CH_4_ production rates. We predicted that that organic substrate quality and nutrient availability, which are reduced by mining and potentially enhanced through restoration, would link to differences in bacterial and archaeal community structure. In particular we predicted that mined sites, relative to natural and restored sites, would have low detectable bacterial and archaeal richness and evenness, concomitant with low rates of greenhouse gas production. Because peat substrate might be a stronger control on community structure than dispersal constraints for prokaryotes, we predicted that land-use effects on community structure within a location would be greater than differences in similar sites across locations.

## Methods

### Study sites, sampling, and peat physicochemical analyses

Canadian peatlands near Rivière du Loup, QC and Shippagan, NB used in the present study were previously described in detail by Basiliko et al. (2007). In each location, we sampled natural sites, actively mined sites, mined sites that had been abandoned for ~30 years and did not have post-harvest peat accumulation, and reflooded block-cut mined sites that had accumulated ~35 cm of *Sphagnum*-dominated peat over ~30 years. Natural and restored sites were dominated by *Sphagnum* moss and shrub vegetation characteristic of bogs or poor fens, and peat in all sites appeared to be dominated by *Sphagnum* remains (Basiliko et al., 2007). The 20–30 and 30–40 cm depth segments were chosen for oxic and anoxic incubations and community analysis. The 30-cm depth was the approximate water-table position at sampling time in the natural, abandoned, and restored sites. Samples were taken in early September and frozen at −20°C and thawed for 3 days at 4°C prior to subsequent analyses. Peat properties characterized by Basiliko et al. (2007) with fresh samples were used in correlation analyses described below. Briefly, microbial biomass and extractable organic C, N, and P, and inorganic N and P were determined using a CHCl_3_-fumigation, K_2_SO_4_ extraction procedure. Peat organic chemistry was characterized using FTIR-spectral analysis to determine the relative concentrations of organic acids or polysaccharides to aromatic molecules, and through differential solvent (diethyl ether and CHCl_3_) extraction of lipids. The humic acid fraction of water-extractable dissolved organic C (DOC) was measured through acid precipitation methods, and the physical degree of humification was measured using the Von Post humification index. Peat moisture content was measured and pH determined in a 4:1 water:peat mixture. Water-extractable inorganic ions (Na^+^, K^+^, Mg^2+^, Ca^2+^, and SO^2−^_4_) were measured using ion chromatography.

### Microbial activity, community structure, and phylogenetic characterization

After thawing, peat was incubated under oxic and anoxic conditions at 20°C to restore microbial activity and standardize temperature and O_2_ availability, and CO_2_ and CH_4_ exchange was measured following methods from Glatzel et al. (2004) and Basiliko et al. (2005). The rate of aerobic CO_2_ production following the final aeration (incubation day 9–10) and the rate of anaerobic CH_4_ and CO_2_ production from day 25 to 30 were chosen to represent aerobic and anaerobic production, respectively and are expressed per g dry peat per day. Immediately following the final gas measurements, DNA was extracted from each of the 48 samples using the FastDNA SPIN Kit for Soil (Qbiogene, Carlsbad, CA, USA) according to the manufacturer's instructions, except that DNA was washed four times with 0.5 ml of guanidine thiocynate (5 M) to remove humic substances (Bengtson et al., 2009). Fragments of genes encoding for bacterial 16S rRNA were amplified from the oxic and anoxic peat DNA using PCR protocols described by Lukow et al. (2000), except that 27f and 1492r PCR primers were used (Preston et al., 2012). Fragments of genes encoding for the alpha-subunit of methanogen-specific methyl-coenzyme M reductase (*mcrA*) and archaeal 16S rRNA genes were amplified from the anoxic peat DNA using protocols from Lueders et al. (2001), and Ramakrishnan et al. (2001). Archaeal 16S rRNA genes could not be amplified from any samples from the mined site at Shippagan and one sample from the mined site at Rivière du Loup. T-RFLP analysis of *mcrA*, bacterial 16S rRNA, and archaeal 16S rRNA gene products followed Ramakrishnan et al. (2001) and Lukow et al. (2000). Relative abundances of individual operational taxonomic units (OTUs) were calculated as the intensities of single peaks larger than 50 bp as a fraction of the sum of all peaks. Peaks with relative intensities less than 1% of the total were removed.

Individual bacterial and archaeal 16S rDNA sequences were isolated from PCR products from anoxic samples with the TOPO TA Cloning Kit (Invitrogen, Carlsbad, CA, USA). Cells from transformed colonies were used in direct-colony PCRs using the included vector-specific M13 primer pair for bacterial 16S rRNA genes or the original 109af-912ar primer pair for archaeal 16S rRNA genes. Ninety-eight bacterial and 138 archaeal PCR products with the correct-size amplification products were purified with the GenElute PCR CleanUp-Kit and sequenced with a 48 lane ABI 377 sequencing instrument (P-E Applied Biosystems, Foster City, CA). Sequence similarity was determined using the contig formation function in SeqMan (Lasergene, DNASTAR, Madison, WI, USA) and in cases where sequences were >98% similar over at least 600 bp length, one sequence was chosen for further phylogenetic analysis For bacteria, the longest sequence with few base-assignment uncertainties was chosen within each >98%-similar contig for further phylogenetic analysis. For archaeal sequences, reverse sequences were also obtained and assembled with the forward sequence. Sequences were deposited in GenBank under the accession numbers JQ934752–JQ934792. Similar sequences from environmental samples and cultured organisms were identified using BLASTN 2.2.21+ searches (Zhang et al., 2000) and included in further analysis. Alignment and phylogenetic tree construction used the MEGA v5 software package with neighbor-joining and maximum composite likelihood methods (Tamura et al., 2011). GeneBank sequences had at least 598 bp of coverage with those from this study. *In silico* T-RFLP analysis was performed on cloned sequences using restriction digest tools in BioEdit Sequence Alignments Editor v 7.0.9.0 (Hall, 1999), and real T-RFs were putatively phylogenetically identified when possible.

### Statistical analyses

T-RFLP data alone (i.e., no clone library data) were used for all community structure analyses. OTU richness, evenness, and Simpson's diversity (Simpson, 1949) indices were calculated. Evenness refers to the pattern of distribution of the individuals between the OTUs and compares the observed Shannon diversity index against an equal distribution of OTUs that would maximize diversity (Krebbs, 1999). Analyses of variance with Tukey *post-hoc* tests were performed on SYSTAT 10 (SPSS Inc. Chicago, IL, USA) to compare inter-site gas fluxes and diversity indices within and between Rivière du Loup and Shippagan. Links between peat properties or CO_2_ and CH_4_ fluxes and diversity indices were explored through Pearson correlation with Bonferroni probabilities. Agglomerative hierarchical cluster analysis (CA) was performed using Ward's method among samples to characterize similarities based on bacterial and archaeal OTU presence and abundance using PC ORD version 4.0 software (MJN Software Design, Gleneden Beach OR, USA). To further examine differences or similarities between sites and samples and to determine if any peat properties, including CO_2_ and CH_4_ fluxes in incubations, potentially described (predicted) any of the variation in the OTU data, Canonical correspondence analysis (CCA) was performed on CANOCO version 4.0 (Microcomputer Power, Ithaca, NY, USA).

## Results

### Phylogenetic characterization of bacteria and archaea

**Figure 1:**
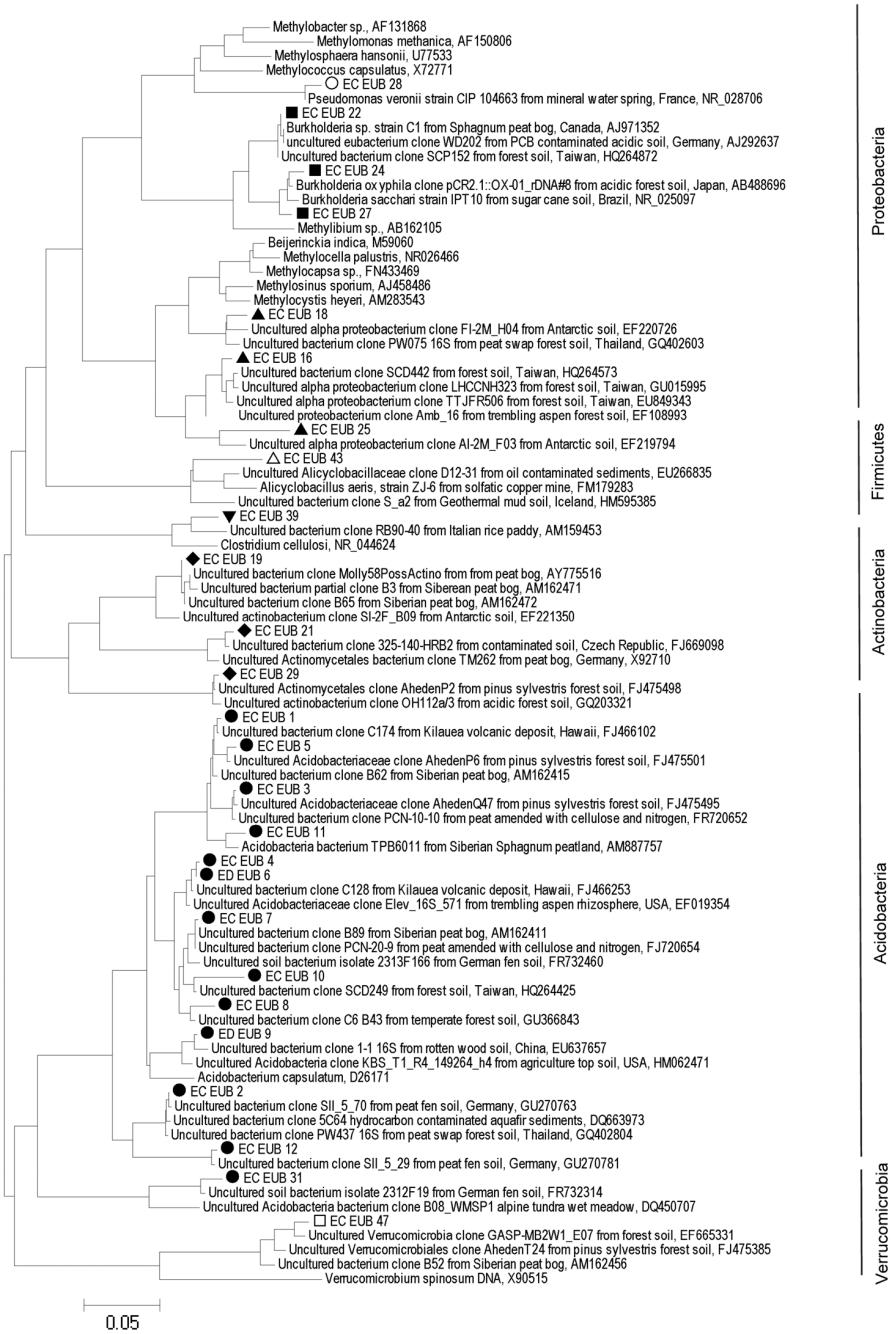
**Bacterial 16S rRNA gene-based phylogenetic tree (neighbor-joining method) of representative sequences retrieved from sites at Rivière du Loup and Shippagan in bold and similar sequences from GenBank**. Distances were computed using the maximum composite likelihood method in the MEGA v5 package. Vertical bars and labels refer to phyla. In reference to Figure 2, sequences with open circles, black squares, and black upward pointing triangles represent classes in the phylum *Proteobacteria*, black circles represent the phylum *Adicobacteria*, black diamonds represent the phylum *Actinobacteria*, open and black upward pointing triangles represent classes in the phylum *Firmicutes*, and open squares represent the phylum *Verrucomicrobia*. Scale bar units are the number of base substitutions per site.

**Figure 2:**
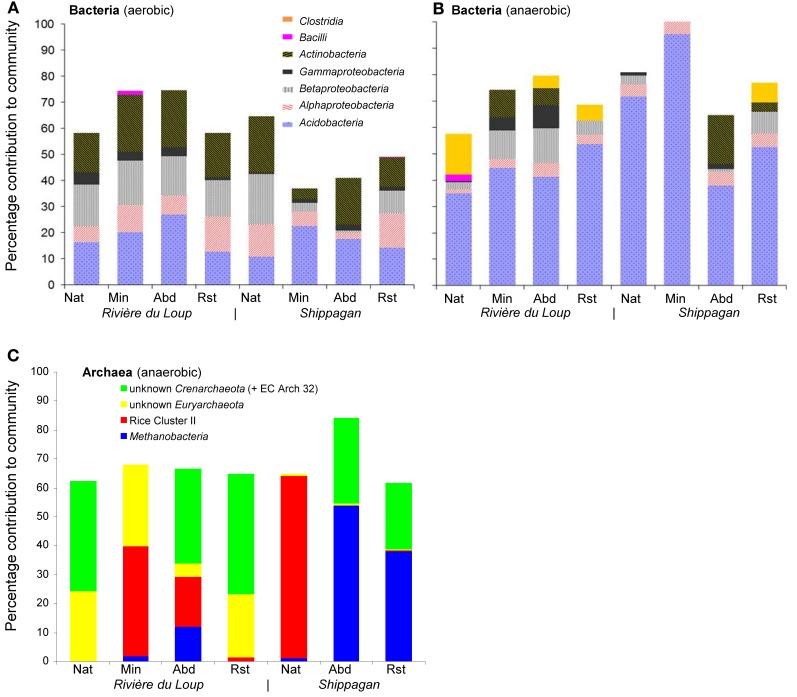
**Bacterial (**A** and **B**) and archaeal (**C**) community compositions (averaged across replicates) based on *in silico* mapping of terminal restriction sites of cloned sequences across natural (Nat), actively mined (Min), mined and abandoned (Abd), and mined and restored (Rst) sites at Rivère du Loup and Shippagan**. Unlabeled portions of each community (i.e., where bars did not add up to 100%) were a result of not being able to assign T-RFs to specific clone library sequences isolated from the anoxic samples.

Bacteria from 5 phyla were detected and many of the sequences were similar to sequences or isolates from other peat environments and mineral soils distributed globally (Dedysh, 2011; Figure 1). Six identical or nearly identical bacterial sequences were found in both Shippagan and Rivière du Loup (Table 1). On average 57 and 75% of T-RFs could be linked to particular clone sequences for oxic and anoxic samples, respectively. These T-RFs represented 7 of 8 encountered classes or phyla (no T-RFs corresponding to *Verrucomicrobia* were greater than 1% of total community peak height); however, there were no apparent land-use, inter-site, or inter-location differences based on *in silico* community composition analyses (Figure 2, Table 1). The T-RFs corresponding to the phylum Acidobacteria represented the dominant OTU in 3 out of 8 sites in the oxic incubations and in all sites in the anoxic incubations (Figure 2). All of the main bacterial groups were represented in both oxic and anoxic samples except the class *Clostridia*, which was not present in oxic samples (Figure 2). In general, the relative abundance of *Actinobacteria* and *Betaproteobacteria* decreased in anoxic compared to oxic samples (Figures 2A,B).

**Table 1:** **Terminal restriction fragment lengths, taxonomic affiliations, and source sites of sequences**.

**Bacteria**				**Archaea**			
**Seq no**.	**T-RF (bp)**	**Taxonomic affiliation**	**Found in:**	**Seq no**.	**T-RF (bp)**	**Taxonomic affiliation**	**Found in:**
1	265	*Acidobacteria*	RDL Abd, SHP Nat	2	490	Rice Cluster II	SHP Nat
2	262	*Acidobacteria*	RDL Nat, RDL Har, SHP Nat, SHP Rst	4	184	unknown *Crenarchaeota*	RDL Nat, RDL Rst
3	262	*Acidobacteria*	RDL Abd, SHP Abd, SHP Rst	6	391	Rice Cluster II	RDL Abd
4	264	*Acidobacteria*	RDL Nat, RDL Rst	8	184	unknown *Crenarchaeota*	RDL Nat
5	261	*Acidobacteria*	RDL Har	9	184	unknown *Crenarchaeota*	RDL Nat, RDL Rst
6	267	*Acidobacteria*	RDL Har, SHP Rst	10	89	*Methanobacteria*	SHP Rst
7	264	*Acidobacteria*	SHP Rst	11	184	unknown *Crenarchaeota*	RDL Nat
8	264	*Acidobacteria*	RDL Abd	12	184	unknown *Crenarchaeota*	RDL Rst
9	149	*Acidobacteria*	SHP Rst	13	184	unknown *Crenarchaeota*	RDL Nat
10	265	*Acidobacteria*	SHP Rst	14	184	unknown *Crenarchaeota*	RDL Rst
11	264	*Acidobacteria*	RDL Nat	17	89	*Methanobacteria*	SHP Rst
12	94	*Acidobacteria*	RDL Adb, RDL Rst	21	184	unknown *Crenarchaeota*	RDL Rst
16	156	*Alphaproteobacteria*	RDL Nat, SHP abd	22	184	unknown *Crenarchaeota*	RDL Nat
18	148	*Alphaproteobacteria*	RDL Rst	31	805	unknown *Euryarchaeota*	RDL Min
19	69	*Actinobacteria*	RDL Har	32	184	unknown *Euryarchaeota*	SHP Rst
21	145	*Actinobacteria*	RDL Abd, SHP Abd				
22	135	*Betaproteobacteria*	RDL Abd, RDL Rst				
24	134	*Betaproteobacteria*	RDL Nat				
25	437	*Alphaproteobacteria*	SPH Abd				
27	140	*Betaproteobacteria*	RDL Har				
28	490	*Gammaproteobacteria*	SHP Nat				
29	134	*Actinobacteria*	SHP Nat				
31	181	*Acidobacteria*	RDL Rst				
39	276	*Clostridia*	RDL Nat, RDL Rst				
43	119	*Bacilli*	RDL Nat				
47	597	*Verrucomicrobia*	RDL Rst				

**Figure 3:**
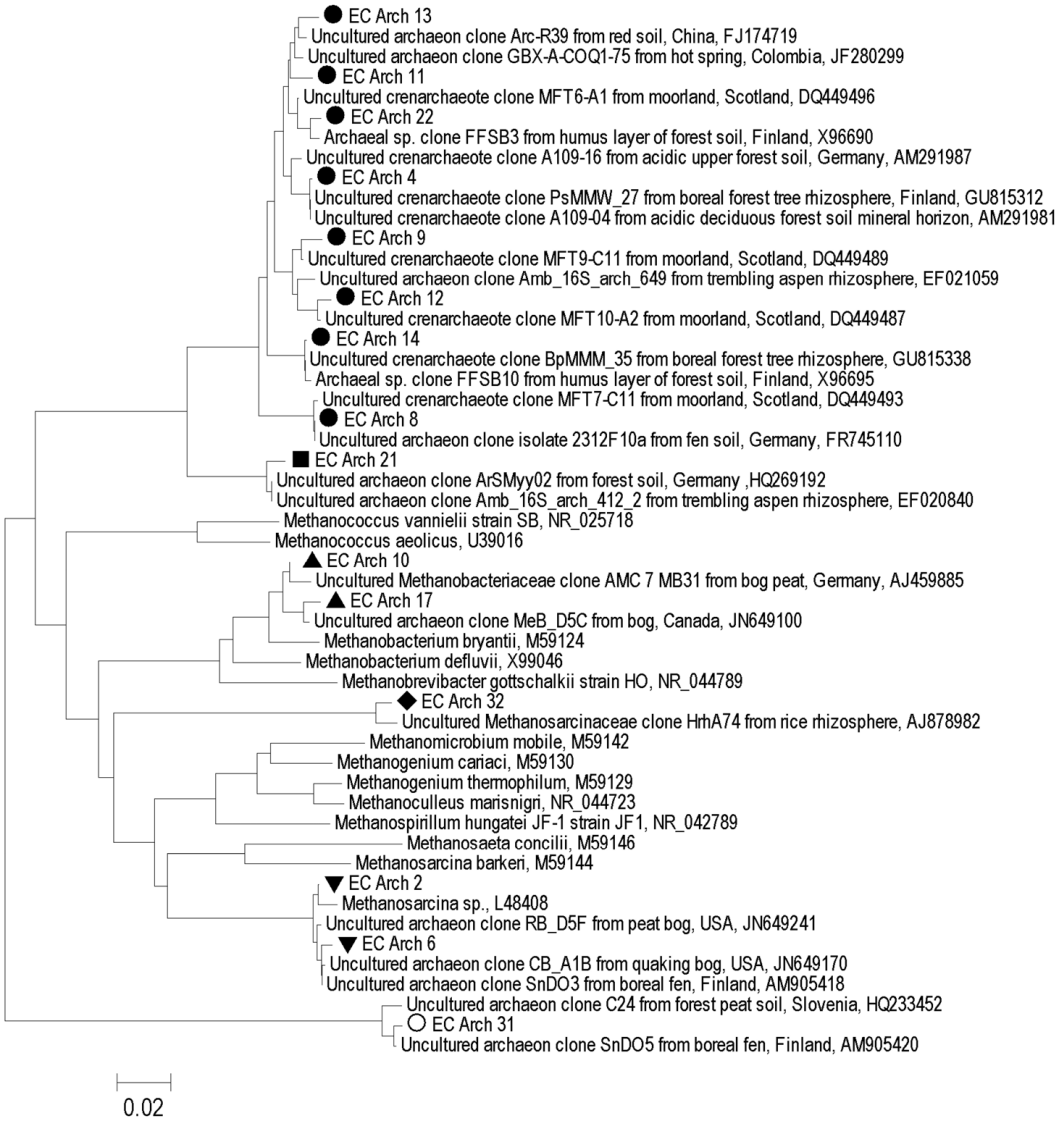
**Archaeal 16S rRNA gene-based phylogenetic tree (neighbor-joining method) of sequences retrieved from sites at Rivière du Loup and Shippagan in bold and similar sequences from GenBank**. Distances were computed using the maximum composite likelihood method in the MEGA v5 package. In reference to Figure 2, black circles represent clones related to unknown *Crenarchaeota* or the single sequence EC Arch 32, open circles and black diamonds represent unknown *Euryarchaeota*, upward pointing black triangles represent the class *Methanobacteria*, and downward pointing black triangles represent Rice Cluster II. Scale bar units are the number of base substitutions per site.

*Euryarchaeota* detected in the anoxic samples were closely related to members of the genus *Methanobacterium* and similar sequences retrieved from North American, Finnish, and German peat soils and rice field soils (Figure 3). Others were related to members of the methanogenic group Rice Cluster II that have previously been detected in North American and UK peat bogs (Hales et al., 1996; Basiliko et al., 2003; Cadillo-Quiroz et al., 2008; Figure 3). Sequences were also retrieved that have no close relatives among described archaea, but are similar to sequences detected in rich field soil, a Finnish fen and a USA mine biofilm (Lu and Conrad, 2005; Baker et al., 2006; Conrad et al., 2008; Juottonen et al., 2008; Figure 3). Crenarchaeotal 16S rRNA gene sequences were closely related to some other sequences detected in soils including moorlands (Jurgens and Saano, 1999; Kemnitz et al., 2007; Nicol et al., 2007; Lesaulnier et al., 2008; Figure 3). No single archaeal T-RF was dominant in most samples; however, unknown *Euryarchaeota* were generally relatively more abundant in the Rivière du Loup sites and the *Methanobacteria* were generally more abundant at the Shippagan sites (Figure 2C). Gene fragments of *mcrA* could only be amplified from the natural and restored samples from Shippagan. Samples from the Shippagan natural site were dominated with a T-RF that corresponded to a group of methanogenic *Euryarchaeota* known as Rice Cluster I with recent isolates from the newly described order *Methanocellales* and the family *Methanobacteriacea* (Lueders et al., 2001), despite our analyses of 16S rRNA gene clone sequences and T-RFLP analysis not detecting the former group. The 237-bp T-RF might also indicate members of Rice Cluster II or other poorly described methanogenic *Euryarchaeota*.

### Land use effects on microbial community and linkages among measured variables

**Figure 4:**
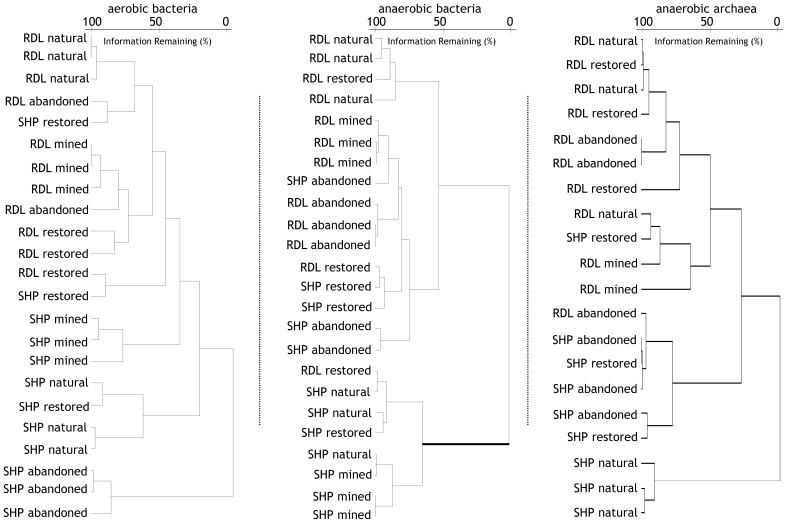
**Hierarchical cluster analysis (calculated with Ward's method; Euclidean distances) of bacterial and archaeal communities in peat from natural, actively mined, once mined and then abandoned, and once mined and then restored sites that had new peat accumulation at Rivière du Loup (RDL) and Shippagan (SHP)**. Operational taxonomic units were defined as unique T-RFs from T-RFLP analysis of 16S rDNA amplified from peat incubated under oxic or anoxic conditions.

**Figure 5:**
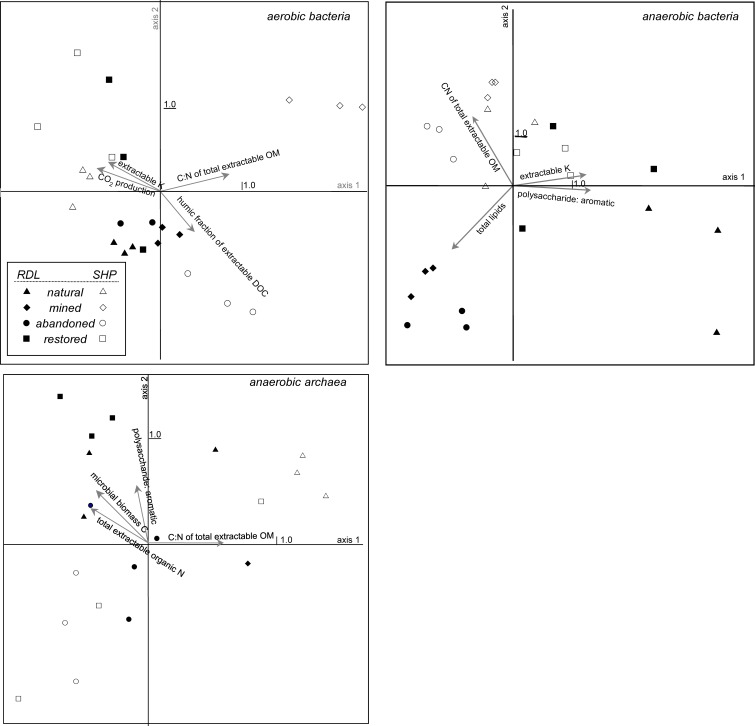
**CCA bi-plot of bacterial and archaeal communities in peat sampled from natural, actively mined, once mined and then abandoned, and once mined and restored mined sites that had new peat accumulation at Rivière du Loup and Shippagan**. Operational taxonomic units were defined as unique T-RFs from T-RFLP analysis of 16S rDNA amplified from peat incubated under oxic or anoxic conditions. Significant peat properties and CO_2_ production correlating most strongly with the first 2 axes, defined by OTU presence and abundances, are indicated with arrows. See Table 4 for additional related canonical correspondence analysis results.

CA and CCA indicated that community structure varied with land-use and between locations (Figures 4, 5, Table 2). In particular, CA of aerobic bacterial taxa grouped most replicate samples together within sites and Rivière du Loup and Shippagan did not have similar community structures (Figure 4). CCA of aerobic bacterial taxa clearly separated mined and abandoned sites from natural and restored sites at Shippagan, indicating that mining and abandonment led to changes in community structure, while restoration returned communities to a state similar more to natural communities (Figure 5). Differences in sites at Rivière du Loup could not be resolved well on the same CCA plot, indicating that community changes through mining and restoration were more pronounced at Shippagan, consistent with CA results. For anaerobic bacterial communities, CA indicated more similarity between sites, likely resulting from substantially increased dominance of *Acidobacteria* in all samples. CCA illustrated clearer differences between sites and locations, indicating both land-use and geographical differences, with the exception of the restored sites that had similar community structure and were clustered together. Although this contrasts with CA results, it is important to note that in defining the axes, CCA down-weighted the importance of the universally-dominant OTU, resulting in less apparent similarity between sites and locations; CCA axes were most heavily defined by members of the class *Gammaproteobacteria* and the phylum *Actinobacteria* in the anoxic incubations. Archaeal communities were largely different between samples from Rivière du Loup and Shippagan based on CA and CCA clustering patterns, however with the exceptions of the replicates at the natural sites clustering together in the CA, restoration did not lead to clear changes in archaeal community structure resolved in CA or CCA.

**Table 2:** **Canonical correspondence analysis results related to Figure 5: (A) inter-set correlation coefficients of variables (CO_2_ production potential and peat properties) correlating significantly and most strongly with the first and second axes and (B) axis summary statistics for aerobic bacteria (1), anaerobic bacteria (2), and anaerobic archaea (3)**.

	**Axis 1**	**Axis 2**	**Axis 3**	**Axis 4**	**Total inertia**
**(A)**					
**Aerobic bacteria**
Humic fraction of DOC	0.4333	−0.5639	0.1017	0.2183	
Water-extractable K^+^	−0.5885	0.4775	0.0733	−0.4254	
C:N of total extractable DOM	0.8605	0.2133	0.1655	0.1512	
CO_2_ production potential	−0.6076	0.4418	−0.0571	−0.2436	
**Anaerobic bacteria**					
Total lipids	−0.6394	−0.7246	0.0091	−0.2104	
Polysaccharide: aromatic	0.8849	−0.0364	0.3875	0.0508	
Water-extractable K^+^	0.8947	0.1093	−0.2826	−0.0232	
C:N of total extractable DOM	−0.4202	0.7629	0.4151	−0.224	
**Archaea**					
Polysaccharide: aromatic	−0.1363	0.4924	0.1029	0.2064	
Microbial biomass C	−0.4522	0.4385	0.1435	0.3374	
Total extractable organic N	−0.5246	0.3478	0.1323	0.2877	
C:N of total extractable DON	0.6648	−0.0204	−0.3333	0.4097	
**(B)**					
**Aerobic bacteria**					
Eigenvalues	0.226	0.163	0.102	0.082	1.195
OTUs-peat property correlations	0.966	0.953	0.956	0.949	
***Cumulative percentage variance of:***					
OTU data	18.9	32.6	41.1	47.9	
OTU-environment relation	31.1	53.5	67.5	78.7	
Sum of all unconstrained eigenvalues					1.195
Sum of all canonical eigenvalues					0.727
**Anaerobic bacteria**					
Eigenvalues	0.303	0.208	0.107	0.061	1.81
OTUs-peat property correlations	0.926	0.955	0.827	0.691	
***Cumulative percentage variance of:***					
OTU data	16.7	28.2	34.2	37.6	
OTU-environment relation	38.1	64.3	77.8	85.5	
Sum of all unconstrained eigenvalues					1.81
Sum of all canonical eigenvalues					0.795
**Archaea**					
Eigenvalues	0.546	0.484	0.445	0.403	5.094
OTUs-peat property correlations	0.923	0.865	0.944	0.857	
***Cumulative percentage variance of:***					
OTU data	10.7	20.2	29	36.9	
OTU-environment relation	21.2	40	57.3	072.9	
Sum of all unconstrained eigenvalues					5.094

Diversity indices generally did not illustrate effects of mining, abandonment, and restoration within Rivière du Loup or Shippagan for aerobic and anaerobic bacterial communities (Table 3). Geographic differences were also not strong. The abandoned site at Rivière du Loup had the greatest archaeal OTU richness, evenness, and Simpson's diversity and had significantly greater values for richness than all other sites. The natural and abandoned sites at Shippagan had significantly greater values for evenness and Simpson's diversity (Table 3). The relationships between peat properties and diversity indices (richness, evenness, Simpson's) were markedly different between oxic and anoxic samples (Table 4). Aerobic bacterial OTU diversity and evenness correlated positively with peat properties characteristic of greater bioavailability, and negatively with properties characteristic of biorecalcitrance. These and similar properties correlated with primary CCA axes. In contrast, diversity and evenness of anaerobic bacteria and archaea were in some cases positively correlated with peat properties characterized as biorecalcitrant by Basiliko et al. (2007) such as the total lipid content or the humic acid fraction of extractable dissolved organic matter. Correlation of properties on CCA axes defined by anaerobic bacterial communities, which separates sites, indicated that diversity in natural and restored sites correlated with peat properties indicative of bioavailability such as the proportion of polysaccharide to aromatic molecules and total extractable K^+^, while diversity in mined and abandoned sites correlated to peat properties indicative of biorecalcitrance (Figure 5, Table 2). Factors that might structure communities therefore appeared to be dependent on land-use, regardless of the location (Rivière du Loup or Shippagan) in which land-use occurred. Anoxic conditions led to less diverse communities dominated by *Acidobacteria*-like organisms, while the bacterial community under oxic conditions was characterized by a more even distribution of taxa. While diversity indices correlated positively to peat properties related to substrate bioavailability and microbial CO_2_ and CH_4_ production, direct correlations between diversity and activity were not significant. Aerobic CO_2_ production correlated significantly with the first two CCA axes defined by aerobic bacterial OTUs, and varied primarily with bacterial communities of samples from natural and restored sites (Figure 5, Table 2).

**Table 3:** **Carbon dioxide and CH_4_ flux potentials and OTU richness, evenness, and Simpson's diversity of microorganisms in peat from Rivière du Loup and Shippagan**.

	**Rivière du Loup**				**Shippagan**			
	**NAT**	**MIN**	**ABD**	**RST**	**NAT**	**MIN**	**ABD**	**RST**
Aerobic CO_2_ production	0.26	0.05	0.05	0.23	0.21	0.09	0.03	0.46
	(0.00)^a^	(0.01)^b^	(0.00)^b^	(0.03)^a^	(0.02)^a^	(0.01)^b^	(0.01)^b^	(0.10)^c^
Bacterial richness	23	21	22	22	19	19	18	19
	(1)^a^	(1)^a^	(6)^a^	(4)^a^	(2)^a^	(1)^a^	(5)^a^	(1)^a^
Bacterial evenness	0.77	0.70	0.70	0.71	0.67	0.69	0.63	0.67
	(0.01)^a^	(0.02)^a,b^	(0.10)^a,b^	(0.04)^a,b^	(0.00)^a,b^	(0.02)^a,b^	(0.06)^b^	(0.00)^a,b^
Simpson's index	17.5	12.2	11.8	13.7	11.0	11.5	8.8	10.4
	(0.6)^a^	(2.1)^a,b^	(5.2)^a,b^	(2.3)^a,b^	(0.8)^b^	(1.3)^b^	(1.7)^b^	(0.3)^b^
Anaerobic CO_2_ production	0.09	0.03	0.03	0.10	0.07	0.07	0.03	0.14
	(0.01)^a,b^	(0.00)^a,c^	(0.01)^a,c^	(0.03)^a,b^	(0.01)^a,c^	(0.00)^a,c^	(0.00)^c^	(0.06)^b^
CH_4_ production	0.01	0.003	0.001	0.480	0.890	0.000	0.001	523
	(0.006)^a,b^	(0.003)^a,b^	(0.001)^a,b^	(0.510)^a^	(0.754)^a^	(0.005)^b^	(0.002)^a,b^	(220)^c^
Bacterial richness	13	15	15	9	7	1	11	10
	(2)^a,b^	(2)^a^	(3)^a^	(1)^a,b^	(3)^b^	(1)^c^	(4)^a,b^	(4)^a,b^
Bacterial evenness	0.54	0.54	0.58	0.42	0.28	0.04	0.50	0.44
	(0.03)^a^	(0.04)^a^	(0.02)^a^	(0.07)^a,b^	(0.12)^b^	(0.06)^c^	(0.06)^a^	(0.13)^a,b^
Simpson's index	5.9	4.8	6.2	3.5	2.0	1.1	5.0	3.7
	(0.9)^a^	(0.8)^a^	(0.3)^a^	(1.1)^a^	(0.7)^b,c^	(0.2)^b,c^	(1.1)^a^	(1.5)^a,c^
Archaeal richness	7	7	12	7	6	n.d.	6	6
	(1)^a^	(1)^a^	(3)^b^	(2)^a^	(2)^a^	n.d.	(1)^a^	(1)^a^
Archaeal evenness	0.36	0.35	0.48	0.33	0.28	n.d.	0.27	0.33
	(0.05)^a,b^	(0.03)^a,b^	(0.02)^b^	(0.03)^a,b^	(0.11)^a^	n.d.	(0.08)^a^	(0.06)^a,b^
Simpson's index	3.5	3.0	4.7	2.9	2.5	n.d.	2.4	3.0
	(1.0)^a,b^	(0.4)^a,b^	(0.1)^b^	(0.4)^a,b^	(1.3)^a^	n.d.	(0.8)^a^	(0.8)^a,b^

Sites were natural (NAT), actively mined (MIN), mined and then abandoned (ABD), and restored trenches at block-cut mined sites that had new peat accumulation (RST). Fluxes are mg CO_2_ g^−1^ peat day^−1^ and μ g CH_4_ g^−1^ peat day^−1^. Operational taxonomic units were defined as unique T-RFs from T-RFLP analysis of bacterial and archaeal 16S rDNA amplified from peat incubated under oxic (bacterial) and anoxic (bacterial and archaeal) conditions. Standard deviations of 3 replicates are in parentheses, and different superscripted letters denote significant differences between sites at P < 0.05.

**Table 4:** **Pearson correlation coefficients between peat properties and OTU richness, evenness, and Simpson's diversity among all sites at Rivière du Loup and Shippagan**.

	**Richness**	**Evenness**	**Simpson's diversity**
**AEROBIC BACTERIA**			
Carboxyl: aromatic	0.459	0.549	0.667
Polysaccharide: aromatic	0.373 (*P* = 0.08)	0.532	0.686
von Post humification index		−0.375 (*P* = 0.07)	−0.484
Microbial C			0.419
Microbial N			0.428
N:P of total extractable DOM			−0.514
**ANAEROBIC BACTERIA**			
Extractable lipids	0.534	0.434	0.486
Humic fraction of DOC	0.576	0.542	0.619
von Post humification index	0.368 (*P* = 0.08)		
Microbial C:N	−0.475	−0.540	−0.398 (*P* = 0.06)
C:N of total extractable DOM	−0.621	−0.618	
Microbial N:P	−0.478	−0.518	
**ARCHAEA**			
Extractable lipids	0.618	0.598	0.524
Humic fraction of DOC	0.508	0.431 (*P* = 0.06)	
von Post humification index	0.509	0.483	0.481
C:N of total extractable DOM		−0.585	−0.512
Microbial N:P		−0.582	−0.468
Na^+^	−0.570	−0.516	−0.396 (*P* = 0.08)
Moisture	−0.560	−0.541	−0.475

Operational taxonomic units were defined as unique T-RFs from T-RFLP analysis of bacterial and archaeal 16S rRNA genes amplified from peat incubated under aerobic (bacterial) and anaerobic (bacterial and archaeal) conditions. Only values with P < 0.10 are included in the table, and unless specified, all relationships are significant (P < 0.05).

## Discussion

### Land use effects on microbial community and linkages among measured variables

Horticultural peat mining and restoration strongly impacts substrate availability (Basiliko et al., 2007) and rates of microbial C mineralization across the eight sites in this study. However, these land-use practices did not consistently affect bacterial or archaeal diversity indices or community composition based on relative proportions of broad phylogenetic groups (phylum and/or class level) of bacteria or archaea. Multivariate analyses based on T-RF-defined OTU presence and abundance could resolve differences between some sites but not clearly across land-uses. Community structure differences across the sites were small compared to overall community structure differences between the 2 locations and between oxic and anoxic incubations. The latter point is somewhat surprising given the relatively short incubation time and known insensitivity of DNA-based T-RFLP approaches (e.g., over rRNA) to detect changes in bacterial communities following flooding (Noll et al., 2005). Although not consistent with our initial predictions, observing only weak effects of land-use change is consistent with studies in other non-mined/restored North American and European bogs and fens, where bacterial community structure was similar across sites that differed in peat physicochemical characteristics and vegetation (Kim et al., 2008; Preston et al., 2012). Across peatlands in the James Bay lowlands region of Canada a similar pattern was also described for archaeal community structure (Preston et al., 2012). Considering that plant communities were similar across the four natural and restored sites in this study, patterns observed for bacterial and archaeal communities contrast with previously reported controls on fungal communities where vegetation was a strong predictor of community structure. In particular, litter chemistry has been shown to play a larger role than water table position in structuring fungal communities (Trinder et al., 2008). Artz et al. (2007) studied a set of mined peatlands, including some that had revegetated a few years previously to others that revegetated >50 years previously, and linked fungal community structure primarily to the successional stage of the plant communities and related chemical differences. It is becoming clear that fungal community structure and function are strongly related to vegetation on previously mined and restoring peatlands (Artz et al., 2007; Trinder et al., 2008, 2009). However, based on the present work, the same patterns are not strong for prokaryotic decomposers.

Although land-use changes across the two locations did not lead to consistent changes in community structure, there were relationships between peat substrate characteristics and bacterial or archaeal community structure across all samples analyzed. That the same factors did not predict high OTU richness or evenness in the oxic vs. anoxic incubations perhaps highlights the importance of water table position and oxygen availability or other short-term conditions over broader land-use related peat substrate changes as a control on microbial community structure. Across other soils types, links between substrate availability and richness or evenness have also been inconsistent (e.g., Nüsslein and Tiedje, 1998; Zhou et al., 2002). In this study we used 4 metrics of characterizing and comparing prokaryote communities based on T-RFLP data (CA, correspondence analysis, single-value diversity metrics, and comparison of proportions of coarse-scale taxonomic groups), and there were some subtle differences in patterns across land-use between the different data analysis techniques. However, differences across location and between aerobic vs. anaerobic communities were more consistent regardless of the metric. This highlights that land-use effects on prokaryotic community structure were indeed small.

### Linking microbial community structure and greenhouse gas production

We had predicted clearer patterns between bacterial and archaeal OTU richness and community structure and rates of CO_2_ and CH_4_ production, as has been reported for methanogens and methanogenesis rates across peatland trophic gradients using T-RFLP (e.g., Godin et al., 2012) and other community fingerprinting techniques (e.g., Basiliko et al., 2003). However, only aerobic CO_2_ production correlated significantly with changes in bacterial community structure elucidated with CCA, but not with any other metric. One potential explanation for the lack of observed relationships might be that we did not target the entire decomposer community, which would have included microfauna and fungi. A second explanation might be that the same taxa can potentially function at a large range of rates across different environments. A third explanation might involve limitations of our community fingerprinting methodology. Although T-RFLP analysis is a widely used rapid microbial community profiling approach that can illustrate differences in community structure of dominant taxa, T-RF-defined OTUs using SSU rDNA might not clearly separate functionally different groups of bacteria or archaea based on rates of CO_2_ or CH_4_ production. Other studies of community structure-activity relationships across peat and mineral soil environmental gradients relying on coarse-scale resolution of defined taxa have reported functional redundancy (Rousk et al., 2009; Myers et al., 2012). Use of high-throughput sequencing approaches might have overcome some of the limitations of T-RFLP analyses, with both lower detection limits and the ability to define OTUs based on partial DNA sequences, rather than restriction fragments that do not necessarily correspond to functional differences among defined taxa.

### Phylogenetic diversity of bacteria and archaea and similarities to other soil environments

Although our relatively small clone library could not identify all taxa identified in the T-RFLP patterns, bacteria identified were generally similar to those isolated from northern peatlands, supporting the hypothesis that constraint on microbial distribution, or the “mass effect”, does not restrict potential community structure, but rather detectable members of a community arise due to local substrate and/or environmental conditions, also known as “species sorting” (Mouquet and Loreau, 2002; Van der Gucht et al., 2007; Andersen et al., 2013). That six bacterial 16S rDNA sequences were the same across the two locations that were >400 km apart also might support species sorting over the mass effect as a driver of bacterial community structure. Across sites and the two locations, members of the phylum *Acidobacteria* often predominated in bacterial communities. *Acidobacteria* are widely distributed soil bacteria capable of growth under acidic and low nutrient concentrations (Ward et al., 2009). Particularly members of the order *Acidobacteriales* have been shown to be dominant community members in Russian and Slovenian peatlands (Dedysh et al., 2006; Kraigher et al., 2006; Ausec et al., 2009; Dedysh, 2011). Some *Acidobacteria* isolated from peat are strictly aerobic (Kulichevskaya et al., 2010) and dominant *in situ* community members under drained conditions (Ausec et al., 2009) in contrast to our findings of increased relative importance under anoxic conditions. *Clostridia*, which were not detected in oxic samples, are known to be obligate anaerobes. That *Actinobacteria* and *Betaproteobacteria* abundance decreased in anoxic (compared to oxic) samples is also consistent with a general, broad understanding of members of these classes in soils (Killham and Prosser, 2007).

Methanogen diversity as detected with T-RFLP analysis and an archaeal 16S rDNA clone library was low across locations and sites compared to other surveys of peatlands and did not include members from the families *Methanosarcinaceaee* and *Methanosaetaceae* capable of growth on acetate found in peatlands elsewhere (Basiliko et al., 2003; Galand et al., 2005a; Cadillo-Quiroz et al., 2008; Godin et al., 2012). This finding is consistent with little to no sedge presence even in the natural or restored sites, as sedges have been previously reported as a key control on acetoclastic methanogenesis (Rooney-Varga et al., 2007; Hines et al., 2008). Obligate or purportedly obligate CO_2_ reducers such as members of the family *Methanomicrobiaceae* or Rice Cluster I (including new isolates of the order *Methanocellales*) common in other peatlands were also not detected. That both methanogen and crenarchaeal sequences were similar to those found in similar soil environments elsewhere gives support to species sorting (i.e., local environmental and substrate characteristics) over the mass effect (i.e., distributional constrains) in structuring the archaeal communities in the peatlands. Our community fingerprinting approach combined the detection of both methanogenic members of the phylum *Euryarchaeota* and non-methanogenic members of the *Crenarchaeota*, which might explain the lack of correlation between archaeal community structure and methanogenesis, particularly as the functional roles of the crenarchaea in peat soils are not yet known. Another factor that might have obscured relationships between the archaeal community and methanogenesis is the occurrence of anaerobic CH_4_ oxidation that would have lowered observed rates (Smemo and Yavitt, 2011). Also, methanogens rely on specific substrates supplied by other microbes. Recent innovative reports by Wüst et al. (2009) and Drake et al. (2009) have demonstrated the importance of trophic interactions between fermenters and methanogens in controlling methanogenesis, factors that were not investigated in this study.

### Summary

Across eight natural, mined, and restored eastern Canadian peatlands, the detected bacteria and archaea were similar to those found in other peatlands and soil environments, although methanogen phylogenetic diversity was relatively low. Despite affecting substrate availability and microbial activity, horticultural peat mining and restoration did not consistently affect bacterial or archaeal diversity indices or community composition, and land-use differences were small compared to those between peat samples from the same site incubated under either oxic or anoxic conditions. Across all samples analyzed regardless of land use, diversity and community structure did correlate with peat substrate and nutrient properties; however, the relationships were not the same under oxic and anoxic conditions and there were no consistent relationships between community structure and activity. Our findings imply that characterizing the bacterial and archaeal community structure might not help understand functional impacts of mining and restoration, as different taxa exhibit functional redundancy and/or the same taxa function at very different rates when exposed to different peat substrates. In contrast to other earlier work focusing on fungal communities across similar mined and restored peatlands, bacterial and archaeal communities appear to be more resistant or resilient to substrate changes brought about by mining and restoration.

### Conflict of interest statement

The authors declare that the research was conducted in the absence of any commercial or financial relationships that could be construed as a potential conflict of interest.
